# Sustainable multifunctional phenolic lipids as potential therapeutics in Dentistry

**DOI:** 10.1038/s41598-022-13292-0

**Published:** 2022-06-03

**Authors:** Naile Dame-Teixeira, Reem El-Gendy, Isabela Monici Silva, Cleonice Andrade Holanda, Andressa Souza de Oliveira, Luiz Antonio Soares Romeiro, Thuy Do

**Affiliations:** 1 Department of Dentistry, School of Health Sciences, University of Brasilia, Campus Universitário Darcy Ribeiro – UnB, Federal District, Asa Norte, Brasilia, DF 70910-900 Brazil; 2 Division of Oral Biology, School of Dentistry, University of Leeds, Leeds, LS9 7TF UK; 3 Department of Pharmacy, School of Health Sciences, University of Brasilia, Federal District, Brasilia, 70910-900 Brazil; 4 Nucleus of Tropical Medicine, School of Medicine, University of Brasilia, Federal District, Brasilia, 70910-900 Brazil; 5 Department of Oral Pathology, Faculty of Dentistry, Suez Canal University, Ismailia, Egypt

**Keywords:** Drug development, Preclinical research, Antimicrobials, Biomedical materials, Regenerative medicine

## Abstract

Phenolic lipids components of the cashew nutshell liquid (CNSL) have molecular structures capable of chemical signalling that regulate gene expression, metabolism and inflammation. This study sets out to assess how CNSL derivatives impact oral bacteria, from an antibacterial and anti-collagenolytic perspective, as well as its biocompatibility with dental pulp stem cells. Two hemi-synthetic saturated CNSL derivative compounds were selected (LDT11-Anacardic Acids-derivative and LDT409-cardanol-derivative). Bacteriostatic activity was tested against *Streptococcus mutans* and *Veillonella parvula.* Antimicrobial capacity against preformed *S. mutans* biofilms was investigated using a collagen-coated Calgary Biofilm Device and confocal microscopy. *Clostridium histolyticum, P. gingivalis* and *S. mutans* biofilms were used to assess anti-collagenolytic activity. Biocompatibility with human dental pulp stromal cells (HDPSCs) was investigated (MTT for viability proportion, LDH assays for cell death rate). LDTs inhibited the bacterial growth, as well as partially inhibited bacterial collagenases in concentrations higher than 5 μg/mL. Dose–response rates of biofilm cell death was observed (LDT11 at 20, 50, 100 μg/mL = 1.0 ± 0.4, 0.7 ± 0.3, 0.6 ± 0.03, respectively). Maximum cytotoxicity was 30%. After 1 week, LDT409 had no HDPSCs death. HDPSCs viability was decreased after 24 h of treatment with LDT11 and LDT409, but recovered at 72 h and showed a massive increase in viability and proliferation after 1 week. LDTs treatment was associated with odontoblast-like morphology. In conclusion, LDT11 multifunctionality and biocompatibility, stimulating dental pulp stem cells proliferation and differentiation, indicates a potential as a bio-based dental material for regenerative Dentistry. Its potential as a bacterial collagenases inhibitor to reduce collagen degradation in root/dentinal caries can be further explored.

## Introduction

In the contemporary global economy, exploring the use of natural resources and maximising their use in medical applications is a topic of great interest to medical and dental research. Recent developments in the field brought focus to plant extracts for oral care purposes. In this context, many Brazilian fruits traditionally used in folk medicine practices have been considered attractive^1^, and may be relevant to the development of functional dental materials. Potential bioactive compounds are phenolic lipids components of the cashew nutshell liquid (CNSL), from *Anacardium occidentale*, a tropical tree native to northeast Brazil. CNSL and its derivatives have increased in value due their discovered qualities as antioxidant^2,3^, antitumor^4,5^, anti-inflammatory^6,7^, antimicrobial^8–13^, between others^14,15^. Cashew tree is widely cultivated in Brazil and its nutshell is considered a by-product and a waste product in agribusiness^2,14^. Most of the health-promoting effects observed in natural CNSL are credited to the presence of the phenolic lipid anacardic acid (AA). AAs, found in *A. occidentale*, are a mixture of compounds that have a salicylic scaffold containing different 15-carbon side chains that can be saturated (n = 0) or unsaturated with one (monoene, n = 1), two (diene, n = 2), or three (triene, n = 3) double bonds.

Among its biological and therapeutic potential as a dental material, it was shown that AAs, as isolated compounds or as a mixture, exert a strong antimicrobial activity against Gram-positive bacteria due to its role as a surfactant, physically disrupting their lipid membranes. This effect may be triggered by inhibition of bacterial enzymes and complexation with metal ions^8,12^. Its activity against *Streptococcus mutans* has been extensively explored, with a consistent high sensitivity at low concentrations^9,12,16^. Due to this strong competence to kill *S. mutans,* AAs have inappropriately been defined as potential “anti-cavities” agents. However, the elimination of *S. mutans* from oral biofilms does not prevent development of caries. Although *S. mutans* presents several virulence factors, such as acidogenicity, aciduricity and production of insoluble extracellular polysaccharides, the oral microbiome holds an abundant functional versatility and resilience. Distinct microorganisms can perform the same metabolic energy-producing functions within a microbial community in a process named functional redundancy^17^, causing caries even in the absence or low abundance of *S. mutans*^18,19^. However, another function of AA compounds that drew the attention of our research group in terms of caries prevention and treatments was the inhibitory effect of matrix metalloproteases (MMP's), particularly MMP's 2, 9 and 14^20,21^.

Several host-derived MMP’s, including the above mentioned MMP-2 and -9, influence the degradation of the collagen matrix during root and dentinal caries lesions progression^22,23^. Bacterial collagenolytic proteases can also be responsible for degrading native collagen^24,25^. Thus, to stop caries progression, it is important to explore novel strategies involving not only the pH buffering, diet control and remineralisation, but also those using anti-collagenase agents. The capacity of AA to inhibit these MMP’s and consequent dentin matrix degradation was previously demonstrated after erosive challenges in vitro^26^. However, there is currently no information about the action of AAs on microbial collagenases. Perhaps the combination of its potential anti-collagenolytic activity and its antimicrobial proprieties may indicate that AA can be used as adjunctive agent to prevent and control several oral diseases.

Nevertheless, for *Anacardium occidentale* extracts or their derivatives to be used in the clinic for prevention or treatment of caries or other oral disease, its cytotoxicity should be elucidated first. Some safety studies have shown positive results with macrophages^6^ and in animal models^27^. Any indication of their use within dental biomaterial requires an assessment of biocompatibility with dental pulp stem cells (DPSC). This study, therefore, sets out to assess how CNSL derivatives—particularly the saturated AA—impact oral bacteria, from an antibacterial and anti-collagenolytic perspective, as well as its biocompatibility with DPSC.

## Results

**Figure 1:**
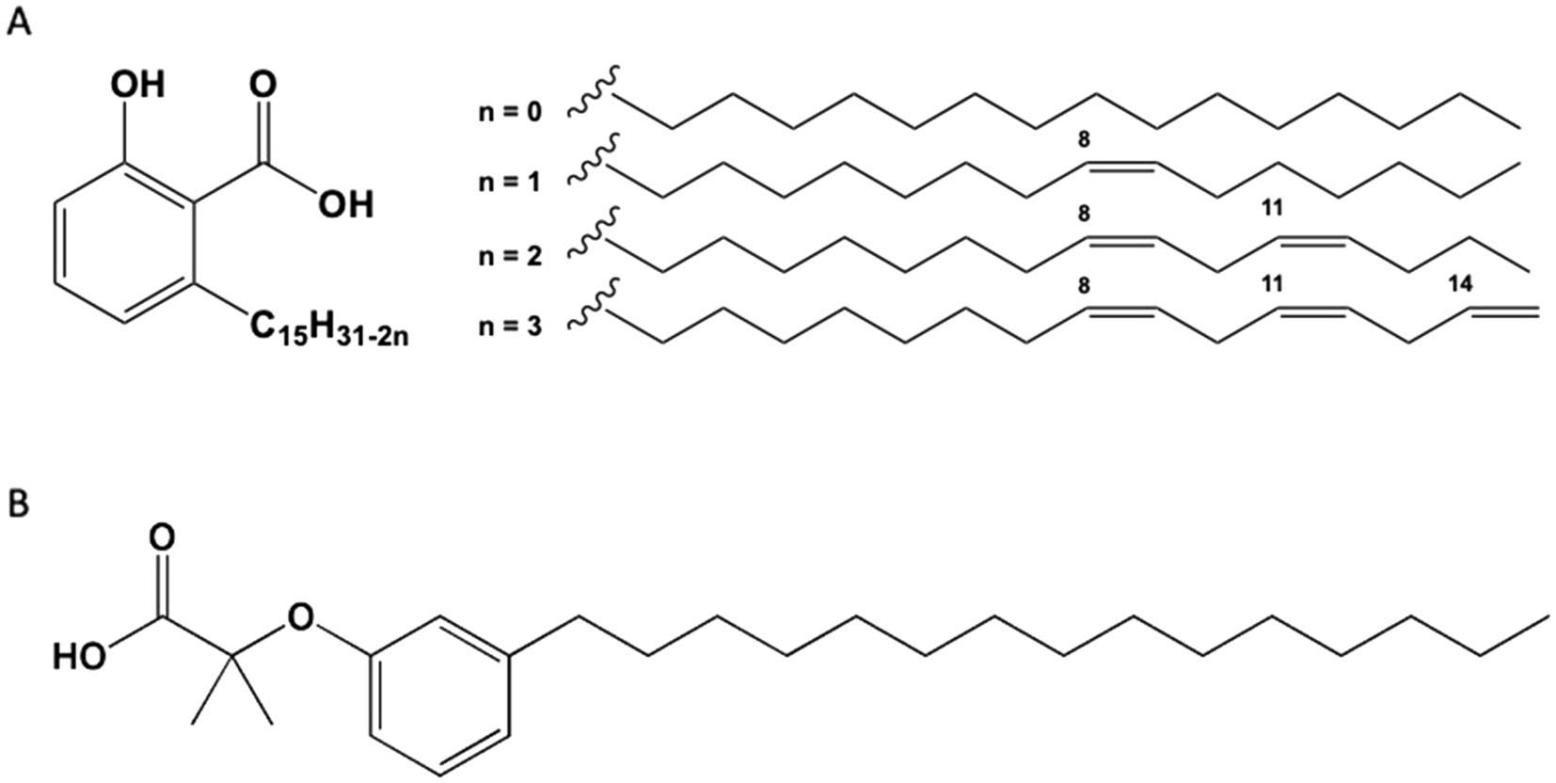
Mixture of anacardic acids (**A**) and cardanol (**B**) present in cashew nutshell liquid (CNSL). LDT11 corresponds to isolated saturated molecules of **A** (n = 0) and LDT409 was synthesized from saturated cardanol (**B**). Details on chemical design and structure are described elsewhere^28^.

Two hemi-synthetic compounds derived from phenolic lipids present in cashew nutshell liquid (CNSL) were provided by the Laboratory of Development of Therapeutic Innovations (LDT) library, from the University of Brasilia: LDT11^6,28^, corresponding to ethanol-soluble saturated molecules of AA (n = 0) and LDT409 synthesized from saturated cardanol (CD)^28^ (Fig. 1).

### Bacteriostatic and antimicrobial activity

To characterise the compounds regarding its bacteriostatic activity, we first selected concentrations previously related to AA (or its derivatives) collagenolytic inhibition. This strategy aimed to achieve the optimal concentration with both, bacteriostatic and anti-bacterial collagenolytic activity. These concentrations were previous defined as efficient to inhibit MMPs 2, 9 and 14 (5, 25 and 34.5 μg/mL = inhibition up to 72% of MMPs; and 69 μg/mL = inhibition of 98% of MMPs)^21^. From that, we increased in 50% the concentrations of the LDT409, based on knowledge that CAs can hold a lower antimicrobial activity when compared to AAs.

**Figure 2:**
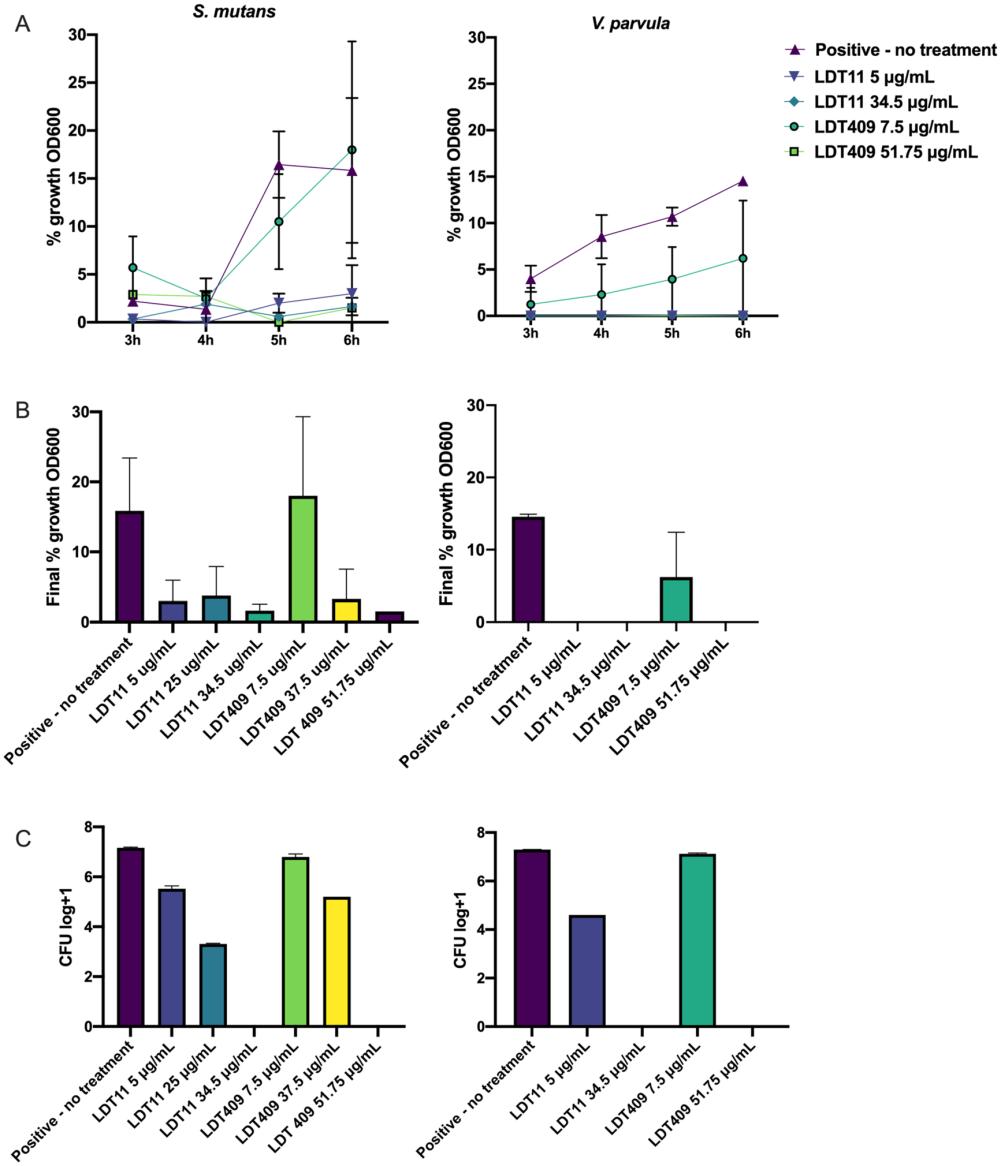
Proportion of planktonic bacterial growth. *Streptococcus mutans* R9 and *Veillonella parvula* ATCC17745 were treated with various concentrations of CNSL-derivatives LDT11 (anacardic acid-derivative) and LDT409 (cardanol-derivative) (OD600). (**A**) Growth curve (% of OD600 growth compared to the baseline immediately after inoculation)—Friedman test, p = 0.003 and 0.002 for *S. mutans* and *V. parvula*, respectively; (**B**) Final proportion of growth after 6 h—Kruskal–Wallis test, NS and p = 0.01, for *S. mutans* and *V. parvula*, respectively; C-CFU (in log + 1) after 6 h of incubation – Kruskal–Wallis test, < 0.0001 and p = 0.003, for *S. mutans* and *V. parvula*, respectively.

The growth of Gram-positive and Gram-negative oral bacteria was inhibited by LDT11 at concentration higher than 5 μg/mL. *V. parvula* seemed to be highly sensitive to this compound and showed total growth inhibition at all tested concentrations. Cells treated with 7.5 μg/mL LDT409 showed consistent growth for *S. mutans* and *V. parvula,* suggesting a slightly lower bacteriostatic activity of CD-derived LDT409 when compared to AA-derived LDT11 (Fig. 2): even in higher concentration (7.5 μg/mL), the LDT409 presented higher bacterial growth than the lowest concentration of LDT11 (5 μg/mL). After 4 h, *V. parvula* growth was lower in the presence of 7.5 μg/mL LDT409 compared to the untreated control.

**Figure 3:**
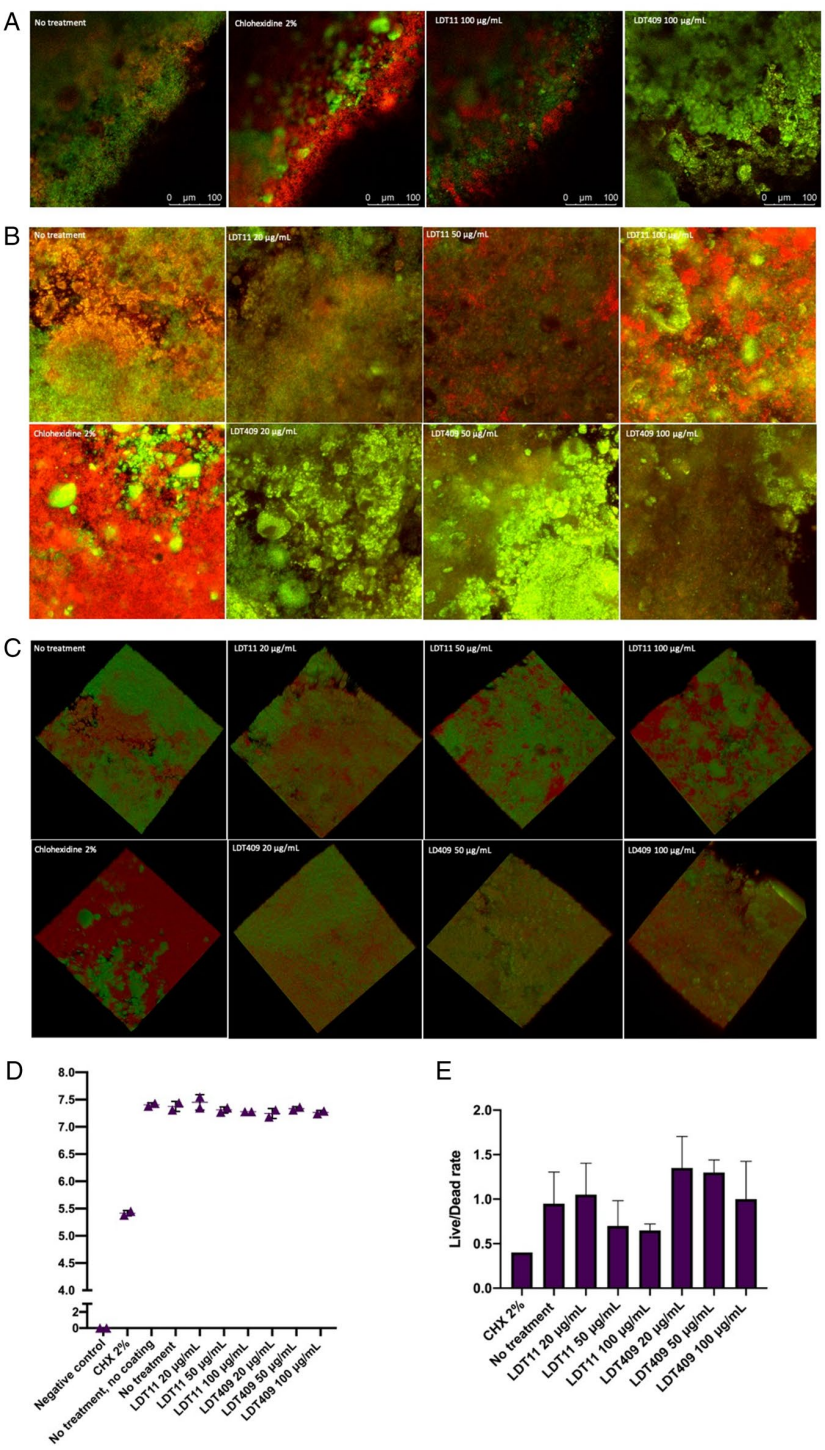
Mature biofilms after treatment with CNSL-derivatives LDT11 (anacardic acid-derivative) and LDT409 (cardanol-derivative). Live/Dead Confocal images in (**A**) 2D biofilms (any point within the biofilm); (**B**) 2D maximum projection, combining all 3D layers; (**C**) 3D biofilm images. (**A–C**) generated using Leica Application Suit X software, v. 3.5.7.23225 (LAS X, https://www.leica-microsystems.com/products/microscope-software/p/leica-las-x-ls/). (**D**) Averages and standard deviation of CFU in log + 1; (**E**) Live/Dead ratio calculated by the area of red and green within the maximum projection images. Kruskal–Wallis results > 0.05.

Superficial layers of the preformed *S. mutans* biofilms were strongly affected by both LDT compounds, as per 3D and 3D confocal images (early biofilms: Supplementary Figure 1; mature biofilms: Fig. 3). Internal areas with dead bacterial cells are observed. The number of colony-forming unity (CFU) was similar between tests and untreated controls, and no viable cells were detected by CFU counts in early biofilms (Supplementary Figure 1, graph), and few in mature biofilms after treatment with chlorhexidine 2% (Fig. 3D). However, there is a dose–response at Live/Dead (L/D) ratio (Fig. 3E). Biofilms treated with chlorhexidine, 50 μg/mL and 10 μg/mL LDT11 presented higher rates of dead cells, and L/D ratio of 100 μg/mL LDT409 was zero. Taken together, the results indicate a slight effect of compounds in concentrations lower than 100 μg/mL onto biofilms’ surfaces, and a better effect of the AA-derivative LDT11 as an antimicrobial against cells in preformed biofilms compared to CD-derivative LDT409.

### Bacterial collagenases inhibition

**Figure 4:**
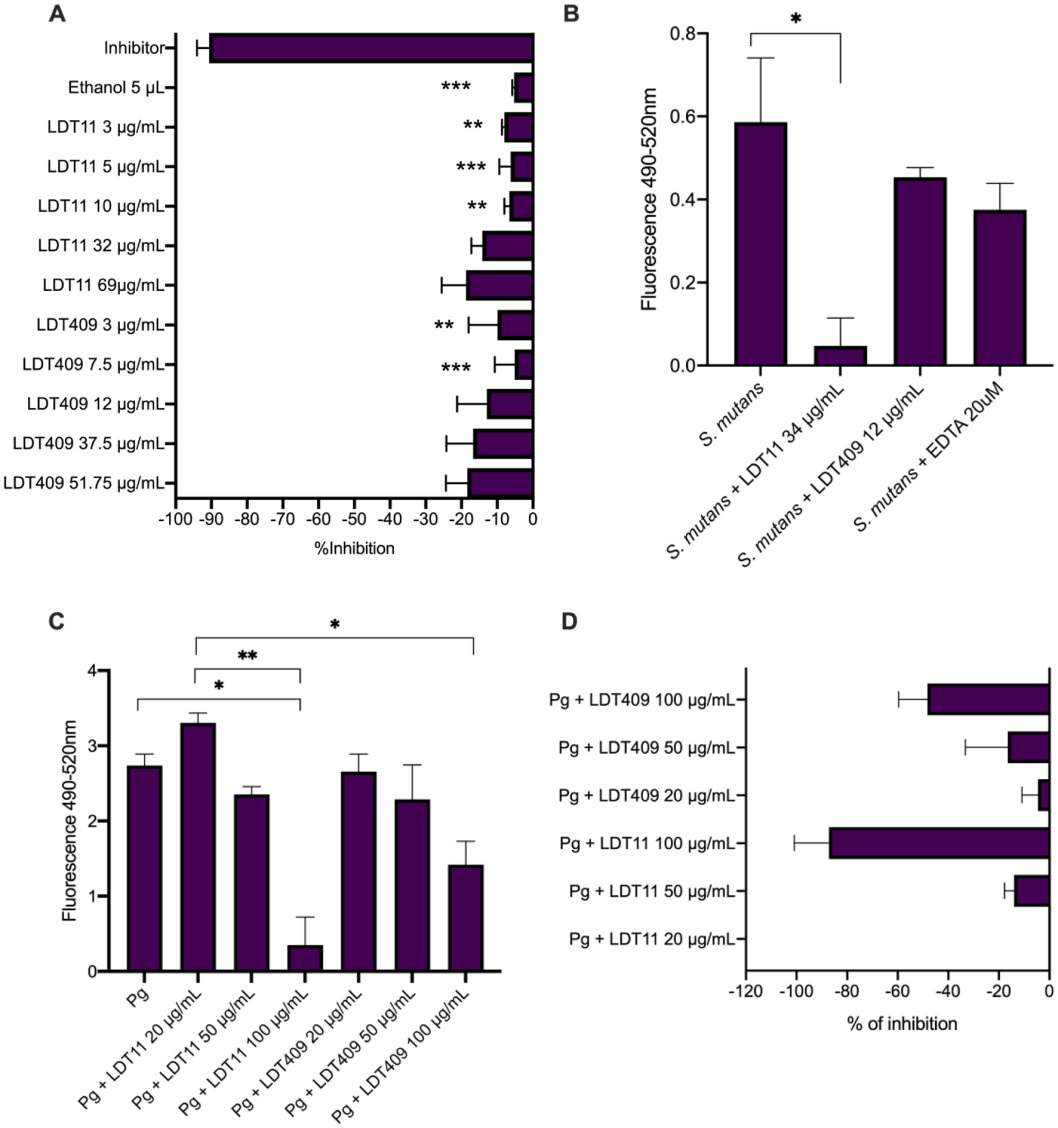
Anti-collagenolytic activity of CNSL-derivatives LDT11 (anacardic acid-derivative) and LDT409 (cardanol-derivative). (**A**) *Clostridium* collagenase inhibition test after 5 min of incubation (substrate = FALGPA, comparisons between tests and the inhibitor control, provided by the supplier). (**B**) Collagenase activity of *S. mutans* mature biofilms (495–515 nm); (**C**) Collagenase activity of *Porphyromonas gingivalis*; (**D**) Proportion of *P. gingivalis* collagenase inhibition in relation to the positive control (untreated cells, same experiment as (**C**). Pg = *Porphyromonas gingivalis.* *p < 0.05 **p < 0.001 ***p < 0.0001; Kruskal–Wallis and Dunn’s multiple comparison test.

We repeated the concentrations from the previous experiment to first test the ability of LDT11 and LDT409 to inhibit *C. histolyticum* of using FALGPA. This collagenase is provided by the supplier and has been extensively used with the purpose of testing inhibitors*.* A significant lower inhibition was observed in all concentrations ≤ 10 μg/mL of both target compounds when compared to the positive control (inhibitor). As can be seen from the Fig. 4A, the maximum inhibition in concentrations between 10–69 μg/mL of LDT11 and LDT409 was 20%, although the substances in test were not compared against each other due to the differences in concentrations.

*S. mutans* biofilms collagenase activity was significantly inhibited by 34 μg/mL LDT11 when compared to the control, however, with the tested concentration of 12 μg/mL LDT409 it was not possible to observe any significant collagenase inhibition in relation to the control (Fig. 4B). To our knowledge, this is the first result on the inhibition of bacterial collagenases in biofilms.

Interestingly, 100 μg/mL LDT11 and LDT409 significant inhibited *P. gingivalis* collagenase activity (Fig. 4C), reaching until 96.8% of inhibition (Fig. 4D). This is an important result, considering the *P. gingivalis* is one of the most relevant proteolytic oral bacteria, and used here as a positive control. A slightly higher inhibitory effect for LDT11 when compared to LDT409 was observed, but it was not statistically significant (p > 0.05). Concentrations ≤ 50 μg/mL also presented approximately 20% of bacterial collagenase inhibition (Fig. 4C). Assuming that LDTs might be altering the pH of the suspension and consequently affecting the results, we checked their pH into the reaction buffer. The buffer itself had a pH = 7.6 (standard deviation-SD = 0.05); while buffer with LDT11 had a pH = 7.11 (SD = 0.09), and that with LDT409 had a pH = 7.04 (SD = 0.15), confirming that the assay pH was neutral.

## Biocompatibility with dental pulp stem cells (DPSCs)

### The Cytotoxic effect on Human dental pulp stromal cells (HDPSCs)

**Figure 5:**
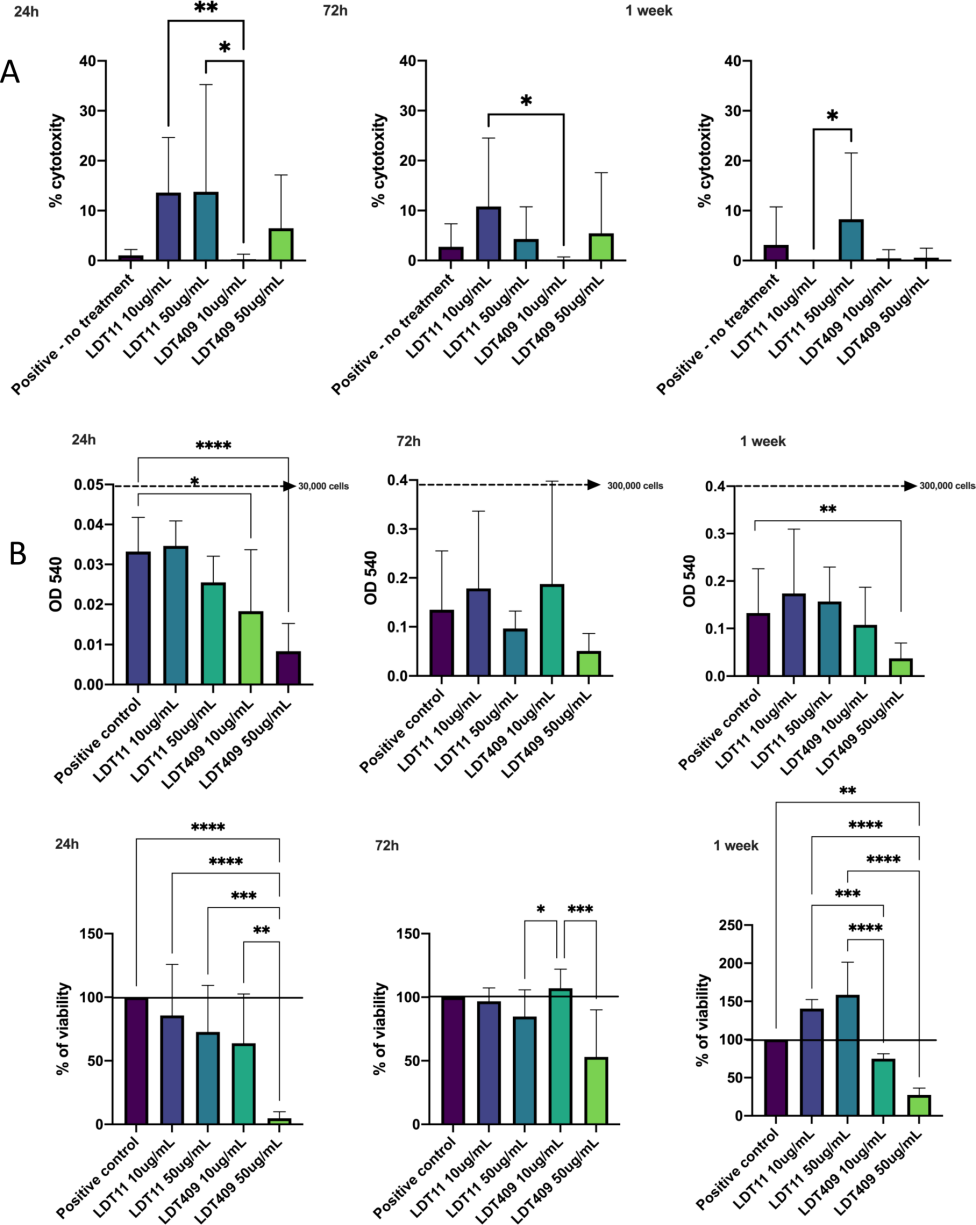
Biocompatibility of CNSL-derivatives LDT11 (anacardic acid-derivative) and LDT409 (cardanol-derivative). (**A**) Percentage of dead cells by LDH assay. Kruskal–Wallis with Dunns's multiple comparisons results are shown as *p < 0.05, **p < 0.01, ***p < 0.001, representing paired comparisons. (**B**) Optical density at 540 nm and the proportion of viability by MTT assay. Dotted line represents the approximate number of cells based on the dose–response curve. The continuous line represents the 100% (cells with no treatment—positive control). Kruskal–Wallis with Dunns's multiple comparisons results are shown as *p < 0.05, **p < 0.01, ***p < 0.001, ****p < 0.0001, representing comparison between the tests and positive control group (untreated cells under basal condition).

Figure 5A shows the proportion of cytotoxicity of LDT11 and LDT409 on DPSCs, measured by the LDH assay (the percentage of cell death). The maximum cytotoxicity of LDT11 and LDT409 was an average of 30% at 10 and 50 μg/mL. After 24 h of incubation, 10 μg/mL of LDT409 had a significantly lower cytotoxicity than LDT11 (10 and 50 μg/mL). Yet, after 72 h of incubation, it seems that the cytotoxicity of LDT11 was reduced, but to levels still higher than LDT409. After 1 week, both concentrations of LDT409 showed almost zero HDPSCs death. LDT409 showed minimal cell death at 10 μg/mL, but a significantly higher cell death rate (10%) of cytotoxicity at 50 μg/mL.

### HDPSCs viability and proliferation in response to LDTs phenolic lipids

For LDT11, cell viability was at its lowest percentage after 24 h but HDPSCs recovered slightly at 72 h and showed a massive increase in viability at 1 week, at both LDT11 concentrations. The cell viability was higher than 100%, confirming HDPSCs proliferation under both tested concentrations of LDT11. A similar trend was observed in HDPSCs treated with LDT409, at 24 h and 72 h at both tested concentrations. However, a significant drop in HDPSCs viability was observed after exposure to 50 μg/mL LDT409 at 24 h (Fig. 5B).

**Figure 6:**
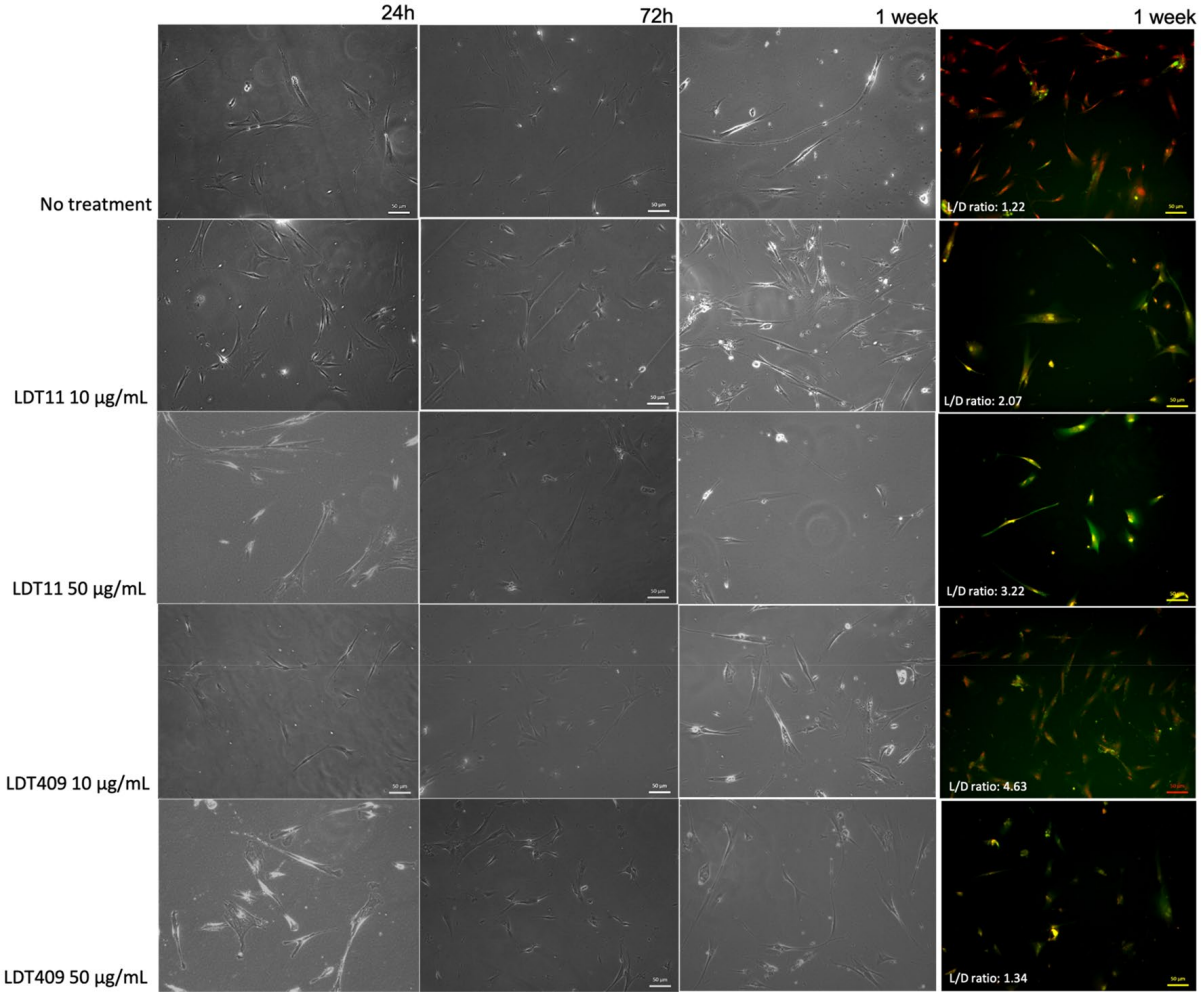
Impact of LDT11 and LDT409 on the morphology and viability of dental pulp stromal cells (HDPSc). Experiments were conducted over 24 h, 72 h, and 1-week using two concentrations of LDT11 and LDT409. HDPSCs were viewed using a light microscope and also stained with Live/Dead staining assay and imaged using fluorescent microscope with merged red and green filters (coloured images on the right side). Images were generated using ZEN 2012 v. 6.1.7601 (https://www.zeiss.com/microscopy/int/products/microscope-software/zen.html).

Cells displayed typical morphological characteristics of stromal stem cells with elongated appearance and fibroblast-like shapes. Odontoblast-like cells were also observed in culture at all time points, and also for the untreated control. However, it seems that LDT11 and LDT409 accelerated the differentiation as more odontoblast-like cells are observed at the 24 h-treated cells, especially at the high concentration (50 µg/mL). Live/Dead staining of HDPSCs after 1 week confirmed cell viability with both types of LDTs, even at the highest concentrations (Fig. 6). The lowest L/D ratio from representative images was from untreated control and 50 μg/mL LDT409.

## Discussion

Natural substances and their derivatives might have prospective biological properties for oral biofilms modulation and can potentially be applied to control either root or dentinal caries. The tested compounds were synthesized from CNSL, which the components are rich in phenolic lipids, including AA and CD mixtures. The AA-derivative compound LDT11 has shown promising multifunctional biological activities as a bio-based renewable dental material, including bacteriostatic, antimicrobial against early and mature *S. mutans* biofilms, and anti-collagenolytic. It also presented great biocompatibility in DPSCs and potential for stimulating cell proliferation. It can, therefore, be relevant for the development of new dental materials for dentinal/root caries treatment, combining sustainability and bioactivity of compounds with a natural origin.

Several AA (isolated compounds or as a mixture) activities were previously recognized, particularly their antibacterial effect. The mechanism behind this activity is mainly linked to its role as a surface-active agent, physically altering the microbial lipid membrane^8^. A role in bacterial respiratory inhibition was also suggested (inhibition of NADH oxidation)^29^. The antimicrobial activity of AAs was also described as being inversely proportional to the length of the C_6_ side chain^8^, and that the alkyl side chain and the degree of its unsaturation are required for enhancing the antibacterial activity against *S. mutans*^11^. Findings are very consistent showing effects of different *A. occidentale* extracts or AA-derivatives against *S. mutans,* in particular strain ATCC 25175^8–12^. Similar results were obtained in this study with the *S. mutans* R9 strain^30^. Although it has been described that the AA antimicrobial activity is limited mainly to Gram-positive bacteria^11,14^, we found strong bacteriostatic activity of LDT11, so as for the CD-derivative LDT409, against *V. parvula,* a Gram-negative anaerobe that comprise the core oral microbiome*.* Likewise, other AA compound derived from *Amphipterygium adstringens* showed antibacterial activity not only against *S. mutans*, but also against *P. gingivalis*^31^.

Nevertheless, most studies evaluated antimicrobial activity of CNSL phenolic lipids in planktonic cells. For its application in the oral cavity, therefore, it is necessary to better explore their effect in biofilms. A preventive effect on biofilm formation was reported for CNSL compounds incorporated into a universal dental adhesive system while its properties as a dental material were preserved, reinforcing the potential of AA for use in clinical practice^32^. Lima et al. (2020) demonstrated some anti-*S. mutans* biofilm activity of zein nanoparticles containing AA when used before the bacterial inoculation as a pre-treatment. However, they found no difference in bacterial viability for mature biofilms treated with their compound^16^. Another study tested the effect of the compound in early biofilm formation, showing a reduction in biomass^13^, however, similarly to the study by Lima et al. (2020), the related "antibiofilm" effect might correspond to the AA potent antimicrobial activity, and not an antibiofilm effect itself as no cells were growing to form biofilms. Our result in preformed biofilms showed a dose–response in bacterial viability, indicating a good antimicrobial effect of LDT11 against mature biofilms in high concentrations. It is important to bear in mind that these results still do not mean that AA can be an anti-caries agent. The oral microbiome resilience and versatility makes it capable of responding to various stressors and, thus, remaining harmless to its host^33^. In the context of the ecological hypothesis of caries, under certain conditions, the balance can be disrupted between members of the microbial community or between the community and the host, predisposing oral sites to diseases development^34^. These conditions include, for example, imbalance in the host diet and an inflammation state, which break the functional stability of the microbial community, leading to dental caries and periodontal diseases, respectively. On the other hand, its anti-collagenolytic potential might be explored for this purpose, while the antimicrobial activity can be explored for other oral diseases.

AA are known to inhibit various enzymes such as urease, glycerol-3-phosphate dehydrogenase, α-glucosidase, lipoxygenases, beta-lactamase, etc.^8,35^. A hypothesis was raised based on the capacity of AAs to inhibit the activity and regulate the expression of MMP’s and gelatinases. It was demonstrated that AAs inhibit the expression of its endogenous activators like MMP-14 and Extracellular Matrix Metallo-Proteinase Inducer, as well as the expression of various components of the Epidermal Growth Factor (EGF) pathway like EGF, Protein Kinase B (Akt) and Mitogen-activated protein kinases (MAPK)^20^. In addition, in vitro dentin erosion was reduced by an inhibition of MMP’s in AA concentrations of 34.4 μg/mL^26^. We also demonstrated activity against bacterial collagenases (AA derived LDT11 was better than CD derived LDT409). Novel approaches for protecting the organic matrix based on protease inhibitors might refine and improve therapies for root and dentin caries^23^. A super-expression of genes encoding U32 proteases of some bacterial species has been indicated. This finding can be a sign of bacterial collagenolytic activity in root caries biofilms, that might be related to functions of collagen degradation during root caries development^25^. In this context, the reduction of activity of bacterial collagenases may indicate a promising role of those substances to control dentinal or root caries. The influence of AA on bacterial collagenases can be explained by its ability to selectively chelate Fe^2^^+^, rendering the metal ions inactive and therefore inhibiting enzymatic activity.

To the best of our knowledge, this is the first study showing the effect of CNSL isolated phenolic biomolecules on DPSCs. Phenolics possessing a C_15_ saturated alkyl side chain were found to be somehow less cytotoxic than the ones with a C_15_ unsaturated alkyl group^5^, which is the case of LDT11. The maximum cytotoxicity of LDT11 and LDT409 was 30% after 24 h of incubation, and did not reach 20% after 72 h. Combining with the results of viability, we considered LDT11 safe in a concentration of 50 μg/mL. The low cytotoxicity of LDT11 against macrophages was previously demonstrated, and the cell viability was greater than 80% at concentrations lower than 50 μM^6^. Other *A. occidentale* AA-rich compounds were biocompatible in human erythrocytes^12^, peripheral blood mononuclear^9^, and other cells lines^36,37^.

The mechanisms behind the biological processes and responses of DPSCs to the target compounds are not totally understood yet. Nevertheless, this may be related to their unique molecular structures capable of chemical signalling which regulates gene expression, metabolism and inflammation in human cells^28^. LDT11 and LDT409 are partial agonists for peroxisome proliferator-activated receptor (PPAR), transcription factors that act as heterodimers, binding directly to DNA. This promotes interaction with co-regulators, modulating the recruitment of basal transcriptional mechanisms and influencing gene expression^28^. Furthermore, AA was an effective inhibitor of the Tip60 histone acetyltransferase activity, responsible for avoiding DNA damage in genotoxic events and involved in cellular differentiation and proliferation^38^. Nonetheless, the present results pave the way for the use of LDT11 on regenerative Dentistry.

The higher cell viability in the presence of increasing concentrations of LDT11 when compared to untreated cells could also be related to AA oxidative damage reduction, which is not present in LDT409. The anti-oxidative role can prevent cell membrane damage and therefore contribute to increased cell differentiation and growth. Also, AA-rich leaf extract of exhibited positive antioxidant and anti-inflammatory properties^39^, and LDT11 significant anti-inflammatory effect has already been demonstrated by the decreased in expression of TNF-α, as well as other interleukins and inflammatory genes^6^. The low cytotoxicity against DPSCs after 72 h and known anti-inflammatory role might particularly be relevant to deep caries lesions treatments. Besides, the inhibition of bacterial collagenases might be of great interest not only for cariology, but also for periodontal treatments. Combined with its anti-inflammatory and antimicrobial potential, the anti-collagenolytic function of AAs might be clinically relevant as adjunctive treatments for periodontal pockets.

Differences in concentrations across experiments can be considered a limitation of this study, however, it was necessary to achieve the optimal concentration in all tested characteristics. Furthermore, the 100 µg/mL LDT11 had the best results against biofilms and collagenases, but it was not tested regarding biocompatibility. This can be a limitation of this study, but we believe that the optimal concentration between 50 and 100 ug/mL will vary depending on the use of the substance. Further experiments include to test LDT11 at concentrations between 50 µg/mL and 100 µg/mL regarding their influence in HDPSCs gene expression and within dental materials, as well as against complex biofilm models.

In conclusion, emerging multifunctional roles of AA-derivative LDT11 from CNSL can be relevant for the development of new dental materials not only for dentinal/root caries treatment, but also for regenerative purposes, combining sustainability and bioactivity of natural compounds.

## Methods

### Growth assays of planktonic oral bacteria

Two representative strains of oral bacteria were characterised, from which their collagenases have been associated with root caries^25^: a Gram-positive (*Streptococcus mutans* R9—this strain was chosen as its sensitivity to AA and CA was not tested yet) and a Gram-negative (*Veillonella parvula* ATCC 17,745 – this bacterium was chosen due to its high proportions in dental biofilms as a part of the caries-associated microbiome). Both strains were obtained from the Division of Oral Biology’s bacterial culture collection (stored in − 80 °C freezer stocks). *S. mutans* was cultured in trypticase soy broth (TSB) (5% carbon dioxide at 37 °C, 48 h). *V. parvula* was cultured in brain heart infusion broth supplemented with 0.6% of sodium lactate (BHIL), anaerobically (85% nitrogen, 5% carbon dioxide, 10% hydrogen) at 37 °C for 72 h. 24 h-subcultures were centrifuged, and the pellets re-suspend into BHI + 1% of sucrose (*S. mutans)* or BHIL (*V. parvula),* and supplemented with LDT compounds (from 5 µg/mL to 51.75 µg/mL), for a final volume of 10 mL. Suspensions were incubated for 6 h and the optical density (OD)600 measured each hour. Aliquots of 6 h-incubated suspensions were used for the Colony Forming Unit (CFU) counting (calculated as log + 1). Positive control (media + inoculum) and negative (plain media) controls were included at the same conditions. Experiments were performed in biological duplicates in independent experiments.

### Antimicrobial activity against early and mature biofilms

The antimicrobial capacity of target compounds against *S. mutans* biofilms was tested using the Calgary Biofilm Device (CBD; MBECTM Assay System, MBEC Biofilms Technology Ltd., Calgary, Alberta, Canada). In order to mimic dentin in the experimental model, CDB pegs were coated overnight with 200 μL of collagen coating solution (Sigma, concentration of 100 μL/0.32 cm^2^), at 25 °C, 65 rpm for 2 h. The pegs were then washed with sterile phosphate-buffered saline (PBS) and treated with human saliva for 5 h (37 °C; 65 rpm). The saliva was previously prepared by adding 2.5 mM DL-dithiothreitol (final concentration 2.5 mM) and 50% of PBS, then sterile filtered^40^.

Pre-cultures of *S. mutans* ATCC 25175 (OD600 = 0.2; 10 μL) were added to the treated CBD wells, with 190 μL TSB. This strain was chosen as a model microorganism due the large amount of data on planktonic minimal inhibitory concentrations on the literature. The plate was incubated at 37 °C under anaerobic conditions, for 24 h (early biofilms) and 7 days (mature biofilms). Fresh TSB was added every 24 h. Concentrations of target compounds ranged from 5 to 50 μg/mL for early biofilms and from 20 to 100 μg/mL for mature biofilms. Chlorhexidine 2% and non-treated pegs were used as controls, as well as pegs without bacteria. In each time point, the pegs were immersed in the formulations (LDTs or controls) for 5 min (37 °C, 60 rpm). CBD pegs (duplicates) were snipped off the plate and washed with PBS to remove loosely adherent bacteria. Each peg was then transferred to 900 μL PBS and biofilms were harvested by scraping with a sterile dental scaler. The collected biofilms were vortex-mixed for 30 s, serially diluted, and inoculated onto BHI agar for the CFU counts. Another couple of pegs were incubated with Filmtracer Live/Dead Biofilm Viability Kit (Molecular Probes, Inc.) for 30 min at room temperature (RT), protected from the light. Samples were imaged using a Confocal Laser Scanning Microscope (Leica Microsystems), using a dry lens of 20 × and 1.5 zoom. 3D images were generated and the maximum projection option applied, combining the biofilm images layers into a 2D image to calculate the area of green and red fluorescence (ImageJ software; RGB measurement tool). Rates of live to dead cells were calculated as:

*Rate of Live/Dead cells* = *area with fluorescence signals by colour threshold green/area in red *(results > 1 means more live cells; < 1 means more dead cells).

## Anti-collagenolytic activity assays

The Collagenase Activity Colorimetric Assay Kit (Sigma-Aldrich, Merck) was used and the protocol followed the supplier’s instructions for collagenase inhibition. This assay uses FALGPA (N-[3-(2-Furyl)acryloyl]-L-leucyl-glycyl-L-prolyl-L-alanine) as the collagenase substrate. Concentrations tested ranged from 3–69 μg/mL, and ethanol negative control (solvent), as well as positive inhibitor control (1,10-Phenanthroline, 1 M, provided by the supplier) were added to the experiment. The provided *Clostridium histolyticum* collagenase was then added, and absorbance (345 nm) was measured immediately, after 5 and 10 min of incubation at RT. The experiments were performed in duplicates. Three independent experiments were performed.

To establish anti-collagenolytic effect against oral bacteria, the *Porphyromonas gingivalis* W3 strain was used as a model microorganism. It was anaerobically cultured in BHI broth (supplemented with hemin and menadione) and assayed by the EnzChek® Gelatinase/Collagenase Assay Kit using the DQ collagen type I as the collagen substrate (from bovine skin, fluorescein conjugate) (Molecular Probes, Inc.), following the supplier protocol. Overnight cultures of *P. gingivalis* (10 mL) were adjust to OD600 = 1.0, pellets washed twice with PBS, and resuspend into 1 mL of the 1 × reaction buffer. The final collagen concentration was 100 μg/mL, and the volume of the bacteria inoculum was 100 μL. The collagenolytic activity of mature biofilms of *S. mutans* (formed into collagen-coated CDB, as described above) were also tested at the same conditions. CBD pegs containing the mature biofilms were dipped into the DQ collagen type I and buffer (with or without LDT11/LDT409) and incubated at RT. *P. gingivalis* cells and *S. mutans* biofilm were incubated in the experiment for 2–24 h, 37 °C. Fluorescence emission from the released fluorescent peptides at the collagen cleaving was read at 495–515 nm.

### Biocompatibility with Human dental pulp stromal Cells (HDPSCs)

#### HDPSCs culture and expansion

Human dental pulp stromal cells (HDPSCs), previously isolated from permanent wisdom teeth from three donors extracted and isolated (Leeds Dental and Skeletal Tissue bank-DREC ethical approval no. 251121/HA/336), were cultured in basal media (Alpha-modified minimum essential medium-α-MEM, supplemented with 20% fetal bovine serum (FBS), 200 mM L-glutamine, and 100 unit/mL penicillin–streptomycin) and incubated at 37 °C, 5% CO_2_. Media was changed each 5–7 days (PBS washing step) until cells reached 80% confluent, where they were detached and passaged as previously described by^41^.

#### Cytotoxicity and viability assays

HDPSCS at P4-P7 were seeded in 48-well plates at a density of 10,000/well and cultured under basal conditions. After 24 h, cells were treated with two concentrations of each compound (LDT11 or LDT409) and maintained in culture for 24 h, 72 h, and 1 week. The compounds, initially reconstituted in ethanol, were diluted in serum free Alfa-MEM with the concentration of ethanol not more than 1%. In each time point, the conditioned media was collected and used for the LDH assay to calculate cell death rate using the Cytotoxicity Detection Kit PLUS (LDH) (Roche), as per the supplier’s protocol. Absorbance was read at 490 nm (Cytation5 reader, serial number 18062215). The cytotoxicity (%) was calculated by the following formula:

$$ \left( {Experimental\; \, value - low\; \, control} \right)/\left( {high \, \;control - low \, \;control} \right) \times 100; $$

where the high control was 100% cell kill and the low control was untreated cells cultured in basal media.

Cell proliferation was assayed by addition of 1 mM MTT (3-(4, 5-dimethylthiazol-2-yl)-2, 5-diphenyltetrazolium bromide) (diluted from 12 mM stock solution into serum free Alfa-MEM). A negative control without cells was added to the experiment, as well as a positive control (untreated cells). Followed by 4 h of incubation in a humidified atmosphere, the formazan purple crystals were washed, solubilized in DMSO, and incubated for 15 min. From each sample, technical replicates of 100 μL were transferred into a 96-well plate and the absorbance was read at 540 nm. Results were calculated from three independent experiments. The viability rates were calculated using the following formula:

$$ {\text{Viability\, rate}} = (test\;OD \times 100)/Positive\; \, control \, \;OD $$

Fluorescence microscopy Live/Dead staining was also performed to confirm and compare HDPSCs viability. Cells were fixed with absolute ethanol, treated with 200 μL of the LIVE/DEAD kit mixture for 30 min/RT, and imaged using the Light/Fluorescent microscope using a 10 × lens (Zeiss microscopy, Carl Zeiss NTS Ltd.).

### Statistical analysis

The proportion of planktonic growth was calculated by (time point OD)–(baseline OD)*100), and compared between groups by the Friedman test and Dunn’s multiple comparison. Results of collagenolytic activity were presented (*P. gingivalis* and *S. mutans* biofilm), as well as proportion of inhibition (*Clostridium* and *P. gingivalis*). Kruskal–Wallis test and Dunn's multiple comparisons test were used for groups comparisons of anti-collagenolytic and biocompatibility assays. Prism v.9.0.2 for Mac was used and the significance level was set at p < 0.05.

## Supplementary Information

Supplementary Information.

## Data Availability

The datasets used and/or analysed during the current study available from the corresponding author on reasonable request.
