# Supplementary material for: Sustainable multifunctional phenolic lipids as potential therapeutics in Dentistry
Source: Sci Rep. 2022 Jun 3;12:9299. doi: 10.1038/s41598-022-13292-0 (PMC9166758; doi:10.1038/s41598-022-13292-0)
Supplement: Supplementary file 1 — Supplementary Information. [file 41598_2022_13292_MOESM1_ESM.docx]

**SUPPLEMENTARY MATERIAL**

**Supplementary Figure 1.** Early biofilms after treatment with CNSL-derivatives LDT11 (anacardic acid-derivative) and LDT409 (cardanol-derivative). The graph represents the CFU in log+1, while images represent the view using Live/Dead Confocal microscopy (2D on the top, 3D on the bottom for the Control and the highest AA concentrations of each tested substance), generated using Leica Application Suit X software, v. 3.5.7.23225 (LAS X, https://www.leica-microsystems.com/products/microscope-software/p/leica-las-x-ls/).
